# Estimating Leaf Area Index in Southeast Alaska: A Comparison of Two Techniques

**DOI:** 10.1371/journal.pone.0077642

**Published:** 2013-11-04

**Authors:** Carolyn A. Eckrich, Elizabeth A. Flaherty, Merav Ben-David

**Affiliations:** 1 Department of Zoology and Physiology, University of Wyoming, Laramie, Wyoming, United States of America; 2 Program in Ecology, University of Wyoming, Laramie, Wyoming, United States of America; 3 Department of Forestry and Natural Resources, Purdue University, West Lafayette, Indiana, United States of America; DOE Pacific Northwest National Laboratory, United States of America

## Abstract

The relationship between canopy structure and light transmission to the forest floor is of particular interest for studying the effects of succession, timber harvest, and silviculture prescriptions on understory plants and trees. Indirect measurements of leaf area index (LAI) estimated using gap fraction analysis with linear and hemispheric sensors have been commonly used to assess radiation interception by the canopy, although the two methods often yield inconsistent results. We compared simultaneously obtained measurements of LAI from a linear ceptometer and digital hemispheric photography in 21 forest stands on Prince of Wales Island, Alaska. We assessed the relationship between these estimates and allometric LAI based on tree diameter at breast height (LAI_DBH_). LAI values measured at 79 stations in thinned, un-thinned controls, old-growth and clearcut stands were highly correlated between the linear sensor (AccuPAR) and hemispheric photography, but the latter was more negatively biased compared to LAI_DBH_. In contrast, AccuPAR values were more similar to LAI_DBH_ in all stands with basal area less than 30 m^2^ha^−1^. Values produced by integrating hemispheric photographs over the zenith angles 0–75° (Ring 5) were highly correlated with those integrated over the zenith angles 0–60° (Ring 4), although the discrepancies between the two measures were significant. On average, the AccuPAR estimates were 53% higher than those derived from Ring 5, with most of the differences in closed canopy stands (unthinned controls and old-growth) and less so in clearcuts. Following typical patterns of canopy closure, AccuPAR LAI values were higher in dense control stands than in old-growth, whereas the opposite was derived from Ring 5 analyses. Based on our results we advocate the preferential use of linear sensors where canopy openness is low, canopies are tall, and leaf distributions are clumped and angles are variable, as is common in the conifer forests of coastal Alaska.

## Introduction

In a variety of ecosystems, overstory leaf area is highly correlated with understory productivity [1]–[4] The relationship between canopy structure and incident light transmission to the forest floor is of particular interest to forest managers studying the effects of succession, timber harvest, and silviculture prescriptions on understory plants and trees [5]–[7]. Following stand initiation, leaf area typically increases until canopy closure stabilizes as the stand matures. Leaf area then slowly decreases as tree mortality leads to a progressive opening of the canopy [8], [9]. The relationship between tree basal area (BA - the area occupied by the trunks of trees in a stand) and canopy cover is curvilinear with little change in the latter with further increases in BA [10]. Understory vegetation tracks these changes in canopy cover, increasing dramatically following removal of the overstory until regenerating trees begin to shade understory plants, virtually eliminating shrubs and herbs. In western conifer forests, canopy closure and elimination of understory plants may be lengthy as shrubs and herbs gradually reestablish only after approximately 150 years in response to improvement in the light environment [5].

Direct estimates of leaf area are difficult to obtain because they are time consuming, labor intensive and undesirable in areas with extremely high, inaccessible canopies such as those found in western conifer forests. Direct measurements can also be fraught with inaccuracies and are only recommended for non-forested ecosystems [11]. In many forest types, where destructive sampling is difficult, leaf area index (LAI) can be predicted from allometric relationships between leaf area and woody stem measurements such as sapwood area or diameter at breast height (DBH) [12]. Alternatively, LAI can be indirectly measured using gap fraction analysis, although such measurements are relatively rare in coastal rainforests [8], [13]. In such environments patterns of LAI are usually inferred from studies in other somewhat similar habitats.

Several tools are available to indirectly measure LAI from beneath the canopy. Commercially available instruments, using various sampling methods, expedite measurements of canopy structure over large areas. These instruments are based on the same model and describe radiation interception by the canopy while assuming a random distribution of foliage. Nonetheless, they vary in sampling techniques, which confer different advantages and disadvantages [14].

Functionally, these instruments can be divided into two classes: linear and hemispheric sensors. Linear sensors (mostly radiometers) measure radiation transmittance over a limited area at one angle of the solar zenith [14]. This measure is analogous to the gap fraction at that zenith angle [15]. Because of the relatively small area measured by linear sensors and heterogeneous light environments, multiple measurements at different locations may be necessary to adequately estimate canopy closure [16], [17].

Hemispheric sensors, such as hemispheric photographs, have a wide field of view (180°) and measure gap fractions at zenith angles 0° to 75° simultaneously [14]. The hemispheric image can be divided into five concentric rings over which LAI is calculated. The number of rings used to calculate LAI can be decreased to improve estimates of canopy closure [15]. This typically involves disregarding the ring closest to the horizon, zenith angles 61–75°, in favor of measuring only a single ring or only the first four annuli, 0–60° [15], [18]. Leblanc and Chen [19], using a Li-Cor LAI-2000 Plant Canopy Analyzer hemispheric sensor near Ottawa, Canada, suggested that the fourth ring (47–58° from zenith) alone is suitable for estimating effective LAI under diffuse light conditions. Several other studies have supported the use of a reduced number of rings in post-processing analysis as long as the foliage angle distribution (G(θ)) is spherical [2], [18], [20].

Previous studies that compared linear and hemispheric techniques in various deciduous and conifer forests have shown that both sensor types produced results that were highly correlated with but biased low relative to direct estimates of LAI [15], [17], [21]. Comparisons among sensor types have yielded varying results. Martens et al. [22], studying orchards and conifer forests in California, found a high correlation between hemispheric photographs and linear ceptometers, but determined that the latter tended to produce higher, more accurate mean estimates than the former. In contrast, Fassnacht et al. [17] found that while estimates from both instruments were highly correlated with direct measurements, the hemispheric LAI-2000 produced higher, more accurate estimates than the linear ceptometer when comparing data from a range of canopy types. Because of this variability in performance, it is useful to identify a single instrument class that provides accurate estimates under a specific canopy type.

Over the past 60 years, large-scale commercial timber harvesting in the Tongass National Forest (TNF) has created over 174,000 ha of young, even-aged forest [23]. The dense regeneration of conifers leads to canopy closure accompanied by a depauperate understory 25–40 years after clearcutting [5]. Understory vegetation is an important component of wildlife habitat as it provides food and cover for many species. Silvicultural thinning involves altering the stand density by removing variable numbers of trees. It has been proposed as a method to accelerate development of late-seral characteristics and improve wildlife habitat in young stands by decreasing LAI and increasing canopy openness [24]–[26]. The majority of thinning in Southeast Alaska is classified as “precommercial thinning” where removed trees are left on site as slash because they have no commercial value [7].

In 2001, the Tongass Wide Young-Growth Studies (TWYGS) were initiated to assess the effects of several thinning treatments on understory plants, overstory trees, and wildlife habitat. Similar studies have been conducted in British Columbia [27], Washington State [28], and Oregon [29], [30] but the TWYGS is the only experiment of this kind in Southeast Alaska. In these studies, canopy closure was only estimated as gap fraction from hemispheric photographs [29] or by using a densiometry index [30] rather than quantifying LAI. Thus, a comparison of linear and hemispheric methods to describe canopy closure in coastal rainforests of the Pacific Northwest has not been conducted yet.

In 2010, we initiated a study to assess the effects of forest thinning treatments on small mammals. Measurements of light filtration to the understory were integral to the investigation of food availability and small mammal responses to these thinning practices. We compared measurements of LAI in 21 forest stands on Prince of Wales Island, Alaska (POW, AK) from a Decagon AccuPAR LP-80 ceptometer (hereafter AccuPAR) [16] and digital hemispheric photography. We evaluated effective LAI estimated by both instruments using simultaneously obtained measurements. Our objectives were to: 1) provide estimates of LAI for each stand type; 2) determine if the instruments produce comparable estimates; and 3) evaluate the performance of each instrument under various canopy conditions by comparing them to values calculated based on non-site-specific allometry. We hypothesized that the two instruments would produce highly correlated estimates of LAI [15], [18], [22]. We also expected LAI to track typical patterns of canopy closure [8] and produce comparable values in stands with similar tree BA. We also tested alternative post-processing practices proposed in the literature for estimating LAI from hemispheric instruments by comparing values integrated over different zenith angles. We hypothesized that LAI estimates from all five rings (Ring 5; zenith angles 0–75°) would be comparable to those from a reduced number of rings (Ring 4; zenith angles 0–60°) and that estimates from Ring 4 would be suitable for use in comparisons with the AccuPAR.

## Materials and Methods

### Ethics Statement

The USDA Forest Service Tongass National Forest and the University of Wyoming approved all field observations and sampling.

### Study Area

**Figure 1:**
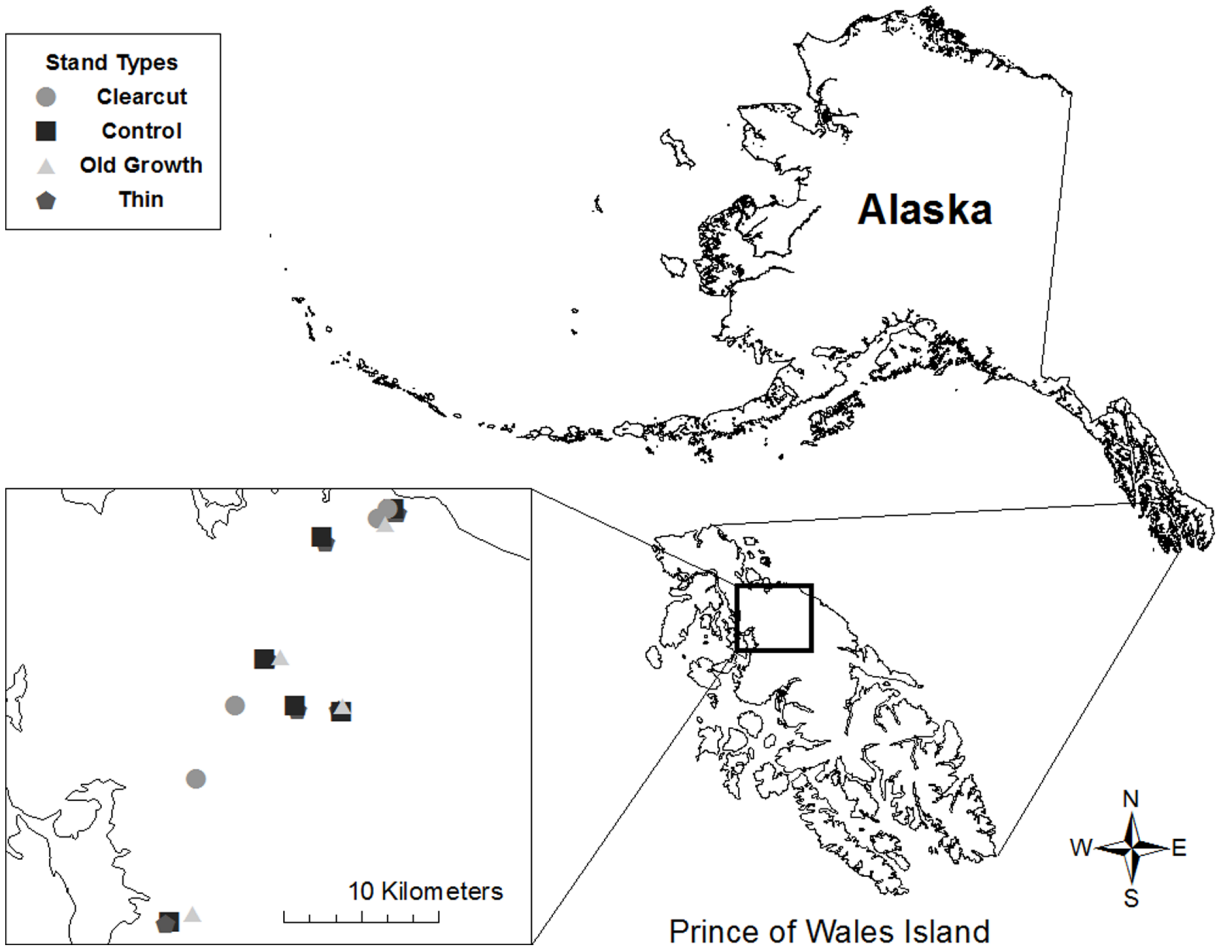
Study area and forest stand locations on Prince of Wales Island, Southeast Alaska. Leaf area index was measured at 79 stations sampled in seven thinned, six unthinned controls, four old-growth and four clearcut stands in late July and early August 2010 and 2011.

The coastal rainforest of Southeast Alaska has a maritime climate. Annual average precipitation on POW, Alaska (55.9° N, 132.9° W; Fig. 1), is 305 cm and average temperatures range from 10–17°C in summer to 0–6°C in winter. Elevation at study sites ranged from 0 to 305 m. Sitka spruce (*Picea sitchensis*) and western hemlock (*Tsuga heterophylla*) are the dominant overstory species at all sites with varying numbers of yellow cedar (*Xanthocyparis nootkatensis*), western red cedar (*Thuja plicata*), and red alder (*Alnus rubra*) interspersed in each stand. The understory consists primarily of *Vaccinium* spp. and false azalea (*Menziesia ferruginea*) with salmonberry (*Rubus spectabilis*), *Ribes* spp. and devil’s club (*Oplopanax horridus*) comprising the remainder of the shrub community. The herbaceous layer is characterized by skunk cabbage (*Lysichiton americanum*), bunchberry (*Cornus canadensis*), false lily of the valley (*Malanthemum dilatatum*), and oak fern (*Cymnocarpium dryopteris*), among various other forbs, mosses and ferns.

We selected six sites across northern POW, coinciding with TWYGS manipulations. The TWYGS comprise a randomized complete block design with replicates of four experimental treatments distributed across the TNF and established from 2002 to 2004. TWYGS sites on POW consisted of an unthinned control stand paired with two stands representing one of the following treatments: (1) moderate (222 trees per acre (TPA) spaced at 14×14 ft; 549 trees per hectare (TPH) spaced at 4.3×4.3 m) and heavy (135 TPA spaced at 18×18 ft; 334 TPH spaced at 5.5×5.5 m) precommercial thinning of 15 to 25 year-old stands; and (2) moderate (170 TPA spaced at 16×16 ft; 420 TPH spaced at 4.9×4.9 m) precommercial thinning combined with two pruning treatments (25 or 50% of the trees were pruned), in 25–35 year-old stands [23]. Retained conifers in each thinned stand were selected based on height, form, vigor, and freedom from disease. TWGYS control stands have little spatial variation and are composed of densely regenerated small-diameter trees. Old-growth sites ranged from low-elevation, high-productivity to high-elevation, mesic sites. A variety of tree size classes contribute to a more heterogeneous canopy in this uneven-aged stand type [5], [8]. Clearcuts ranged in age from 1–12 years post-harvest, contained few remaining overstory trees, and were characterized by a dense layer of residual timber slash mixed with regenerating shrubs and tree seedlings. Our study sites included seven TWYGS thinned treatment stands, six TWYGS unthinned (control) stands, four old-growth and four clearcut stands (for a total of 21 stands). This sampling design encompasses a wide range of stand ages and tree size classes, tree composition and densities, and canopy types across POW.

### Sampling Design

LAI derived from each instrument is defined here as the effective LAI (L_e_), or plant area index (PAI), because it is related to the total solar radiation intercepted without distinguishing between woody and photosynthetically active material [15]. L_e_ for non-flat leaves is defined as one-half the total stand leaf area (or interception area) per unit of ground surface area [31]. Therefore, it provides an estimate of the amount of radiation intercepted by canopy elements such as conifer needles, which is essential to indirectly estimate LAI, and it relates the interception area of a canopy to the ground area beneath.

All forest stands were sampled in summers of 2010 and 2011. For below-canopy measurements we randomly selected four stations in each stand from the 40–52 small mammal trapping stations established in a grid format at 25 m intervals. Measurements from each instrument were obtained at the same time and in virtually the same position at each station. We preferentially collected canopy measurements during overcast conditions. However, some sampling was conducted under partially cloudy or direct sunlight conditions due to logistical constraints.

### AccuPAR Ceptometer

To obtain an estimate of L_e_, we measured photosynthetically active radiation (PAR) above and below the tree canopy. Below-canopy PAR (PAR_b_) was measured while holding the AccuPAR level at the lowest possible point above understory vegetation (∼1.5 m). Ten measurements were recorded at the center point, 10 m out in each cardinal direction, and once again at the center for a total of 60 measurements per station. Each measurement is an average of the 80 PAR photodiodes along the AccuPAR wand. The 10 m-radius circle measured with the AccuPAR is comparable to the canopy area represented by each hemispheric photograph. Simultaneous above-canopy PAR (PAR_a_) and solar zenith angle measurements were obtained by placing a second AccuPAR that logged data every minute [16] in a nearby, unshaded clearing.

The ratio of PAR_a_ to PAR_b_ (τ) was used to calculate L_e_ by inverting the equation for predicting scattered and transmitted PAR proposed by J. Norman [16]. We only included measurements in which the beam fraction (the ratio of direct beam radiation from the sun and radiation from all other sources) [16] was 0.01. Due to local variations in sky conditions, PAR_a_ sometimes exceeded PAR_b_, leading to negative estimates of L_e_ at stations under large canopy gaps or in clearcuts. We excluded all such negative estimates from the dataset. We used an average of the total L_e_ estimates per station in subsequent analyses.

### Digital Hemispheric Photography

Hemispheric photographs were taken with a Nikon Coolpix S560 10 mega-pixel digital camera (Nikon Inc., Melville, NY, USA) that was mounted on a 1 m tripod with the lens facing skyward and the camera body leveled. A full fisheye lens (Digital King, M.Power, Japan) was then magnetically attached to the lens barrel. We used an umbrella to protect the camera and lens during periods of precipitation. We positioned the tripod as close to the station center point as possible while avoiding overtopping understory vegetation. An automatic setting (normal mode, 3648×2736 pixels, 1∶8 compression) was used and three canopy pictures were taken at each station while coinciding measurements were taken with the AccuPAR.

The digital images were downloaded directly from the camera to a personal computer. One photo per station was chosen for subsequent analysis based on image quality and color contrast. The photographs were analyzed for L_e_ using the gap light analyzer (GLA), Version 2.0, image processing software package [32]. L_e_ was estimated over the zenith angles 0 to 60° (LAI 4 Ring) and 0 to 75° (LAI 5 Ring) [32], which are automatically provided by the GLA software.

Gap fraction is acquired from images by estimating the fraction of exposed sky in each pixel. A threshold level (hereafter “thresholding”) of light must be specified to classify pixels as “sky” or “foliage.” Manual thresholding techniques introduce observer bias because the threshold level is based solely on the users’ best judgment [15], [33], [34]. Manual thresholding is not only subjective but is time consuming and labor intensive. Therefore, we first converted all photos to black and white (binary) images using the blue channel and following the automatic thresholding technique described in Nobis and Hunziker [35] and implemented using the software SideLook (ver. 1.1.01; www.appleco.ch) [36].

We then introduced the binary images into the GLA software and analyzed them using site-specific configuration settings (i.e., location and elevation). Because we failed to note the magnetic north when taking the pictures in the field, we analyzed each image with north in eight azimuthal orientations to avoid bias. The eight estimates of L_e_ from Ring 4 and Ring 5 were averaged separately for each station.

### Tree Basal Area (BA)

Concurrent with sampling of canopy cover, we randomly selected 9 stations in each stand from the 40–52 small mammal trapping stations for vegetation sampling. DBH was measured for all live trees with ≥2.5 cm at breast height in a 0.03 ha (10 m radius) plot. We then calculated the BA of each plot by dividing the summed BA value of individual trees by the plot area (in hectares). Seventy-five stations at which LAI was sampled had a paired BA estimate for comparison.

### Allometric Estimates

Leaf area index was predicted based on the allometric relationship between DBH and foliar biomass (FB):
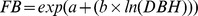
where FB is given in kilogram per tree and *a* and *b* are species-specific coefficients obtained from the literature [37]–[40]. We applied this non-site-specific equation to all trees within each vegetation plot to calculate the FB of individual trees. We then used a species-specific half-surface “specific leaf area” value to convert from foliar biomass to projected leaf area, LAI_DBH_ [37]–[40]. Correction factors were applied to western hemlock and western red cedar calculations [40] but were unavailable for the species-specific equations used for all other trees. The LAI_DBH_ value of individual trees was summed and divided by the plot area. We generated an allometric LAI value for all stations at which LAI and BA were sampled.

### Statistical Analyses

Of the 84 total stations sampled, we removed five from the final analysis. Images for three of these stations were lost during data transfer and two stations yielded negative AccuPAR L_e_ estimates. The remaining 79 stations were stratified into groups based on stand type (thinned, control, old-growth, clearcut).

We used ordinary least squares regression to assess the relationship between AccuPAR and hemispheric photography measures of L_e_. Univariate models were created to compare the AccuPAR to hemispheric photography estimates first using data from all five rings (Ring 5) and then using data from only the first four rings (Ring 4). We also used linear regression to model the relationship between Ring 4 and Ring 5. We tested the data for normality using normal probability plots of standardized residuals. Model residuals were plotted against predicted values to test for goodness-of-fit (GOF) followed by a non-constant error variance (NCV) test [41]. Models with non-constant error variance result in a significant p-value (i.e. α <0.05).

We then assessed the performance of the AccuPAR and hemispheric photography by stand type using paired t-tests [42] as well as by evaluating the overlap of the 95% confidence intervals. We used similar methods to explore the performance of the two methods in detecting differences among the three thinning treatments (14×14, 16×16 and 18×18 ft).

We used nonlinear regression to assess the relationship between BA and L_e_ measures derived from the AccuPAR and hemispheric photography. We constructed second-order, univariate models for BA versus AccuPAR and BA to hemispheric photography estimates. We used ordinary least squares regression and a univariate model to assess the relationship between BA and LAI_DBH_. We used paired t-tests to compare LAI_DBH_ values to L_e_ estimates. Because LAI_DBH_ is known to overestimate true LAI in mature stands [12], [39], [40], we restricted comparisons with other instruments to stations where BA was less than 30 m^2^ha^−1^. We tested the data for normality using normal probability plots of standardized residuals followed by GOF and NCV tests. All statistical analyses were performed using Program R and the “car” package [41], [43].

## Results

### Comparing Ring 4 to Ring 5

**Figure 2:**
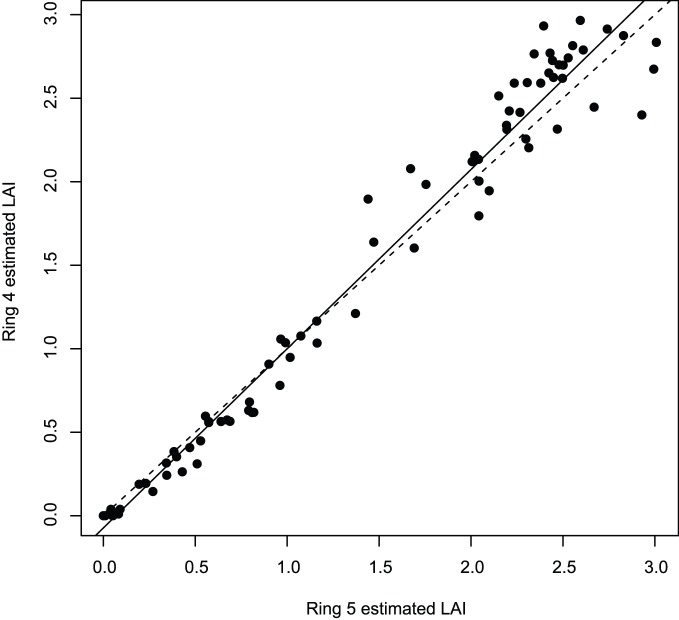
Using five annuli versus four annuli. Comparison of the LAI estimates from hemispheric photography using all five annuli (Ring 5) and using only the first four annuli (Ring 4). The dashed line is the 1∶1 relationship; the solid line is the regression.

Station averages of L_e_ produced by integrating over the zenith angles 0–75° (Ring 5) were highly correlated to those integrated over the zenith angles 0–60° (Ring 4; *r = *0.98, *p*<0.001; Fig. 2). Despite this agreement, there was scatter in the data and Ring 4 tended to produce higher estimates of L_e_, especially at higher values. Discrepancies between the two measures were significant and caused the model to fail the GOF and NCV (*p*<0.01) tests. A normal probability plot also indicated that this model violated the assumption of normality. The indication that the relationship between Ring 4 and Ring 5 is not linear implies that the foliage angle distribution (G(θ)) in this forest type is neither constant nor spherical.

The GOF test suggested that there was a slight trend (i.e. heteroscedasticity) in Ring 4 vs. AccuPAR model residuals. However, the non-constant error variance test indicated that this violation was not significant (*p* = 0.58). The normal probability plot showed that the Ring 4 model strayed from normality and suffered from kurtosis. Neither logarithmic nor square root transformations alleviated heteroscedasticity in the residuals. We found no pattern in residuals of the Ring 5 vs. AccuPAR model based on GOF and NCV tests. The normal probability plot indicated that the Ring 5 data was normally distributed, suggesting that using a linear model to compare L_e_ between the AccuPAR and Ring 5 was valid. Therefore, we used only Ring 5 data for further analysis.

### Instrument Comparison

**Figure 3:**
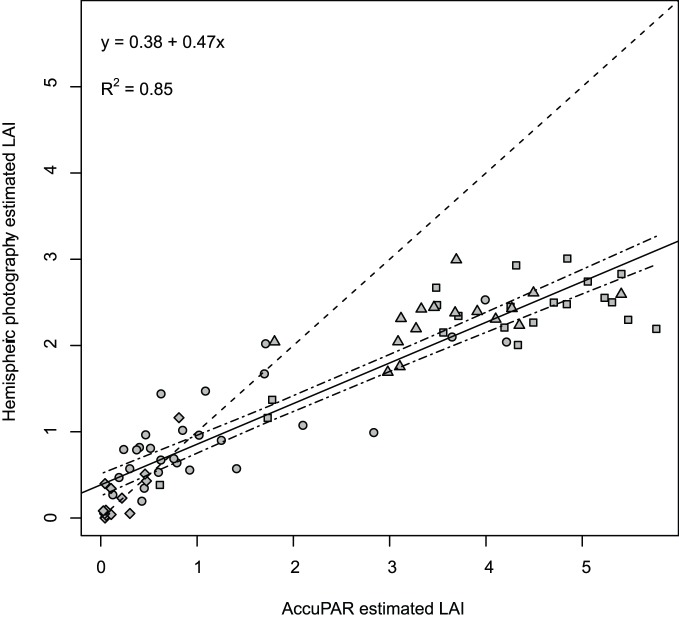
Evaluating LAI estimates from the AccuPAR Ceptometer and hemispheric photography. Comparison of matched LAI estimates from the AccuPAR Ceptometer and those derived from hemispheric photography (Ring 5) by stand type for 79 samples in 21 stands on Prince of Wales Island, Alaska. Circles represent thinned stands, squares control stands, triangles represent old-growth, and diamonds denote clearcuts. The dashed line is the 1∶1 relationship; the solid line is the regression.

**Figure 4:**
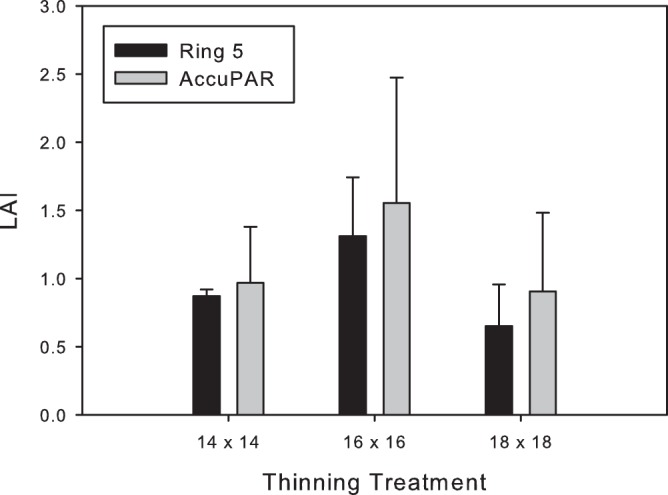
Mean L_e_ estimates in thinned stands. Means (±95% confidence intervals) for L_e_ estimates obtained from AccuPAR and hemispheric photographs (Ring 5) from four randomly selected stations in each of two stands thinned to 14×14 (ft), two thinned 18×18, and three stands with a 16×16 spacing on Prince of Wales island, Alaska in summers 2010 and 2011. Both techniques yielded a similar pattern indicating higher L_e_ estimates in 16×16 treatments likely represent the older age of the stand at the time of thinning. AccuPAR measurements appear slightly higher and more variable than those obtained by hemispheric photographs.

L_e_ estimates ranged from 0.02 to 5.8 for the AccuPAR, and from 0.0 to 3.0 for the hemispheric photographs. In general, station averages of L_e_ produced using the AccuPAR were strongly correlated with results obtained using Ring 5 (*r* = 0.92; Fig. 3). However, the slope of the regression line was significantly different from 1 and the strength of the correlation varied markedly when the dataset was stratified into stand types (Table 1). On average, the AccuPAR estimates were 53% higher than the L_e_ derived from Ring 5 except in clearcut stands. Measures of L_e_ were better correlated in thinned and control stands (*r* = 0.80 and 0.82, respectively) than in old-growth stands (*r* = 0.55), but showed the highest correlation in clearcuts (*r* = 0.86). Although the pattern was similar for L_e_ estimates for the three thinning treatments, AccuPAR values were slightly higher and more variable than Ring 5 estimates (Fig. 4).

**Table 1:** Means (95% confidence intervals) for LAI_DBH_ and L_e_ estimates obtained from AccuPAR and hemispheric photographs (Ring 5) from 21 forest stands on Prince of Wales Island, Alaska in summers 2010 and 2011.

Stand Type	*n*	LAI_DBH_	AccuPAR	Ring 5	Coefficient	Effect size	*P*-value
Thinned	28	2.88 (1.94–3.83)	1.20 (0.77–1.63)	1.00 (0.77–1.22)	0.80	0.20	0.17
Control	21	8.35 (6.26–10.44)	4.12 (3.55–4.70)	2.26 (1.20–2.53)	0.82	0.82	<0.01
Old-Growth	16	17.32 (13.49–21.15)	3.63 (3.23–4.03)	2.30 (2.15–2.46)	0.55	0.58	<0.01
Clearcut	14	0.18 (0.06–0.30)	0.20 (0.08–0.32)	0.24 (0.08–0.41)	0.86	−0.17	0.32
All	79	7.13 (5.42–8.85)	2.29 (1.88–2.71)	1.47 (1.25–1.68)	0.92	0.36	<0.01

For each stand type, the number of paired measurements (*n*), the correlation coefficient (*r*) between the two sensors, and statistical difference (paired t-test at α = 0.05) are presented.

### LAI and Basal Area

Diagnostic tests of residuals indicated slight violations of assumptions in the BA vs. AccuPAR model. Models were then re-run using log-transformed estimates of BA. This transformation removed all violations but resulted in seven negative values. Because these values are biologically nonsensical, we removed them from the analysis.

**Figure 5:**
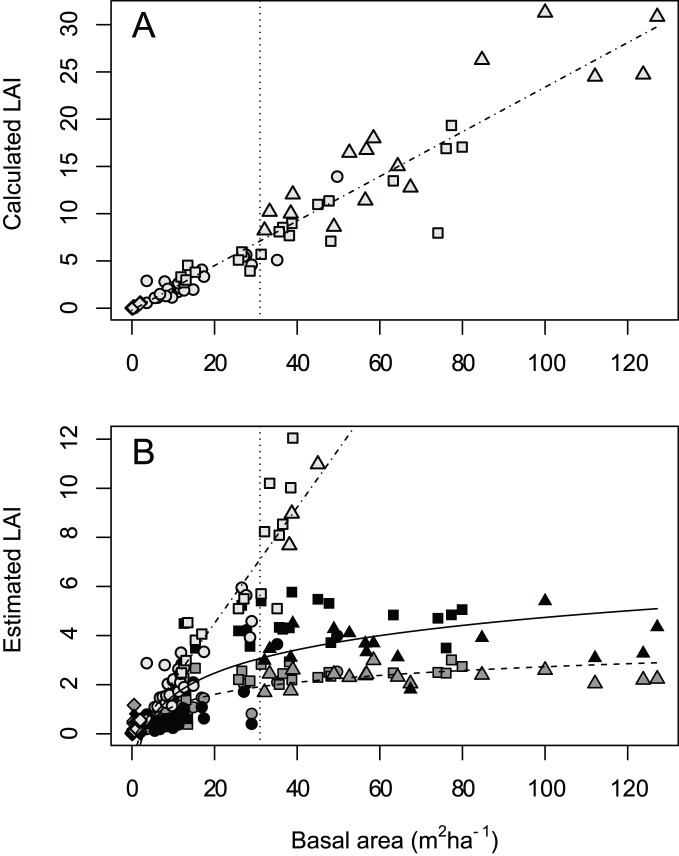
LAI and tree basal area. (A) The relationship of tree basal area and allometric LAI based on tree diameter at breast height (LAI_DBH_). (B) The relationship of tree basal area and LAI estimates from the AccuPAR (solid line, black characters), hemispheric photographs (dashed line, dark gray characters), and LAI_DBH_ (dot-dash line, light gray characters) on Prince of Wales Island, Alaska. Circles represent thinned stands, squares control stands, triangles represent old-growth, and diamonds denote clearcuts.

L_e_ estimates from the AccuPAR and hemispheric photographs were significantly related to BA (*R* ^2^ = 0.61 and 0.73, respectively and *r* = 0.78 and 0.85, respectively) based on a logarithmic transformation of basal area. The AccuPAR produced higher L_e_ estimates than hemispheric photographs across the entire range of BA (Fig. 5). In general, there was a rise in L_e_ as BA increased from clearcuts to old-growth stands, reaching a near asymptote at 30 m^2^ha^−1^ (Fig. 5).

LAI_DBH_ estimates ranged from 0.008 to 31.29 and were on average 207% and 370% higher for the full range of data than AccuPAR and hemispheric photographs L_e_, respectively (Fig. 5). For stations with BA less than 30 m^2^ha^−1^, LAI_DBH_ was significantly different from L_e_ estimates from the AccuPAR and hemispheric photographs (p<0.001). The mean of the differences was 0.95 (95% CI: 0.58–1.32) for the AccuPAR and 1.28 (0.90–1.66) for hemispheric photographs.

## Discussion

Our results clearly show that in the coastal rainforests of Southeast Alaska, hemispheric photography may yield biased estimates of LAI as evident from the discrepancy between these measurements and calculated LAI_DBH_. We suspect that the linear sensor better captured LAI estimates because of the nonrandom spatial distribution of foliage of western conifers, the non-spherical angle [G(θ)] of needles, and the variable height of the canopy in these forests. In addition, these same factors likely also led to the lower reliability of LAI estimates when we reduced the number of rings in the analysis of hemispheric photographs.

Our results demonstrated that effective LAI estimates from the two post-processing techniques (Ring 4 and Ring 5) were highly correlated, but showed increasing scatter at higher LAI values. Under direct sunlight conditions, scattering of light increases diffuse radiation, causing a decrease in effective LAI [17]–[19]. This effect can be magnified at large zenith angles [15]. During conversion to binary images, small gaps at large zenith angles may be missed, resulting in underestimation of gap fraction [34], although there is little evidence that this effect caused much of the variation in our dataset. In addition, the height of the canopy, especially in old-growth stands, may increase multiple scattering of light within the canopy and concomitantly increase the penumbral or shadowing effect [15]. Thus, it is possible that because of these phenomena, Ring 4 values would be more sensitive to sky conditions than Ring 5 estimates. This is in contrast with conclusions by Leblanc and Chen [19], who found that Ring 4 estimates were less sensitive to sky conditions in broad-leaf and pine forests in Ontario. Alternatively, a strong agreement between the two post-processing techniques would have indicated that the foliage angle, G(θ), was constant, which would correspond to a spherical foliage angle distribution [18]. When this condition is met, reducing the number of rings should not yield bias in LAI [20]. Indeed, studies using a reduced number of rings have shown that deviations from a spherical foliage angle are uncommon in conifer forests in northern Wisconsin and western Montana, or in mixed deciduous-conifer forests in Ontario and boreal forests in the Yukon [17]–[19]. However, Schleppi et al. [44] found large variation in G(θ) at forested sites in Switzerland. When G(θ) is not constant, the elimination of rings will result in errors in LAI estimates that fluctuate in magnitude with leaf angle [20], [45]. Because of the potential sensitivity of Ring 4 estimates to sky conditions and the lack of consistency in G(θ), we advocate using all five annuli for LAI estimates in this forest type.

Uniformly diffuse conditions are optimal for hemispheric photography [34]. These conditions provide the highest contrast between foliage and sky [14] and usually occur when skies are overcast or at dawn or dusk. Direct illumination of the canopy leads to a scattering effect, a corona effect around the sun, reflections, and color blurring [15], [34], yielding an underestimation of LAI. In contrast, the AccuPAR provides reliable estimates under a wider range of environmental conditions [45], [46]. Although we preferentially collected both measurements under uniformly overcast skies, some images were taken during partially cloudy or direct sunlight conditions. Using a digital camera capable of capturing high quality, full size images [34], [47], and using the blue channel and automatic thresholding [35] during processing, we were able to alleviate some of the effects of direct sunlight on the canopy in our analysis. However, our inability to avoid these conditions could explain some of the low estimates produced by hemispheric photographs. It is interesting that we observed high correspondence between the two methods in clearcut and thinned stands, even in days with partial overcast conditions, suggesting that the main bias in LAI estimates with hemispheric photography resulted from the behavior of the canopy. Despite this correspondence, the AccuPAR captured more of the variability of thinned stands caused by the differences between thinning treatments. Nonetheless, we found that the AccuPAR could be more sensitive in such open canopies. Even under completely overcast skies, local variations in conditions sometimes caused PAR_b_ to exceed PAR_a_, resulting in unusable LAI estimates. This highlights the importance of correct placement of the second, unshaded AccuPAR.

In addition to the effects of sky condition, gap fraction models used to indirectly measure LAI assume randomness in the spatial distribution of foliage. In coniferous forests, the grouping of needles on shoots represents a serious violation of the random distribution assumption. Clumping at all scales will result in canopy transmittance greater than predicted by the random model. Failure to account for foliage clumping can lead to underestimates of LAI [12], [14], [33]. We were unable to eliminate the effect of foliage clumping on measurements obtained from hemispheric photographs. A correction factor has been proposed for use with the LAI-2000 [14],[17], although its applicability to hemispheric photography is unclear. Alternatively, it may be possible to estimate LAI separately for each of the five rings in hemispheric photography. Such analysis may alleviate the effects of clumping and render the assumption of random distribution valid.

In contrast, use of the AccuPAR has the potential to eliminate the effect of foliage clumping by employing the finite-length averaging method developed by Lang and Yuequin [48]. By linearly averaging over small spatial scales where the assumption of randomness is more valid, this procedure reduces the effect of clumping. Using the length of the probe (0.8 m) as the finite transect length and the average of the eight readings measured along the probe, we eliminated the effect of clumping at scales larger than 0.8 m [15]. This contributes to greater values of LAI than those obtained from hemispheric photography, which still include the effect of clumping. It is difficult to assess the proportion of the clumping effect removed by using finite-length averaging with AccuPAR measurements. Therefore, these data may still be biased low because of clumping at scales smaller than the averaging length.

The main difference between the two methods in our dataset occurred in the stands characterized by more closed canopies. The ability to resolve fine-scale architecture of canopies is a necessary requirement for accurate measurements of gap fraction in both methods. Several studies found that hemispheric photography produces inaccurate measurements of LAI under dense canopies with less than 10% openness [34], [35], [49]. This may explain the lack of agreement between the instruments in unthinned control stands. The unthinned stands we measured were in the “doghair” stage and were extremely dense with canopy cover typically exceeding 90% [5]. Our estimates from hemispheric photography in this stand type were low in comparison with the AccuPAR and LAI_DBH_ as well as with values of LAI for this stand type in other studies [8]. Sitka spruce stands of similar age in Ireland and comparable BA (15–59 m^2^ha^−1^) in Scotland were characterized by an average LAI of 6.5 and 6.3, respectively [39], [49]. These measurements support the conclusion that the higher AccuPAR estimates for this stand type in our study were more representative.

That the AccuPAR measurements were less biased is also evident from the comparison with LAI_DBH_. However, the allometric algorithm we used failed to capture the reduction in LAI as stands progress from immature to old-growth stands and increase in basal area. This is consistent with previous work that has shown that allometric equations based only on DBH yield unrealistically high estimates in mature stands where DBH exceeds 100 cm [12], [39], [40]. As trees develop, growth is largely concentrated on increasing crown width and depth and therefore leaf area. As stands reach maturity, crown width and possibly depth stabilize due to constraints imposed by water transport, competition, spacing, and shading. It is therefore unreasonable to expect the continuation of an exponential relationship between DBH and LAI in such mature stands [40]. Alternative algorithms that incorporate additional canopy characteristics such as openness and crown depth would have likely improved our estimates of LAI_DBH_ [39]. These parameters were not included in our vegetation surveys but should be incorporated into future studies in this region.

Hemispheric photography failed to produce LAI estimates that follow the typical pattern of canopy closure under tall, old-growth canopies. The old-growth stands in our study area are characterized by large western hemlock and Sitka spruce trees and these conifers at times may exceed 40 m in height [5]. Frazer et al. [8] found that LAI decreased as stands progressed from immature, closed canopy to open, old-growth stands. This pattern was clearly captured by the AccuPAR measurements but not by the photographs. Indeed, Chen et al. [15] also found that hemispheric instruments overestimated LAI in tall stands due to the disappearance of small canopy gaps in shadows.

Additional support for the values produced by the AccuPAR can be derived from comparison with other studies. Estimates of LAI from indirect measurements have ranged from 1.46–4.22 along the shorelines of western Prince William Sound, AK [13], 2.04–5.44 on Vancouver Island, British Columbia [8], and from 3.0–4.0 on the Oregon coast [50]. This is especially striking when considering the relationship between LAI measurements and BA. Hennon et al. [10] described an asymptotic relationship between BA and canopy cover in wet yellow cedar habitats on Baranof and Chichagof Islands in Southeast Alaska. Our analyses revealed a similar pattern, which was more pronounced with the AccuPAR L_e_ measurements than with values generated from the hemispheric photography.

## Conclusions

Based on our findings, we believe that our L_e_ estimates obtained from the AccuPAR better reflect the canopy characteristics of these forests and suggest that measuring LAI in the coastal rainforests of the Pacific Northwest is best conducted with linear sensors. Nonetheless, linear instruments are less available commercially, are expensive, may require prior training, and do not provide a permanent image of the canopy that can be used for monitoring through time. Thus, when logistical and operational constraints prevent the use of a linear sensor, with some considerations, hemispheric sensors may be an acceptable alternative. In relatively open canopies with lower scattering of blue light, hemispheric photography may yield unbiased and accurate data. In closed canopies, however, this technique should be used with caution, potentially after a correction factor has been developed [19]. In summary, despite the limitations of linear sensors listed above, based on our results we advocate their preferential use where canopy openness is low, canopies are tall, and leaf distributions are clumped and angles are variable.

## References

[1] Gray AN, Spies TA (1996) Gap size, within-gap position and canopy structure effects on conifer seedling establishment. J Ecol 84: 635–645 10.2307/2261327

[2] Jelaska SD, Antonić O, Božić M, Križan J, Kušan V (2006) Responses of forest herbs to available understory light measured with hemispherical photographs in silver fir–beech forest in Croatia. Ecol Model 194: 209–218 10.1016/j.ecolmodel.2005.10.013

[3] Klinka K, Chen H, Wang Q, De Montigny L (1996) Forest canopies and their influence on understory vegetation in early-seral stands on west Vancouver Island. Northwest Science, 70(3), 193–200.

[4] Wright EF, Coates KD, Canham CD, Bartemucci P (1998) Species variability in growth response to light across climatic regions in northwestern British Columbia. Can J For Res 28: 871–886 10.1139/x98-055

[5] Alaback PB (1982) Dynamics of understory biomass in sitka spruce-western hemlock forests of southeast alaska. Ecology 63: 1932–1948 10.2307/1940131

[6] Canham CD, Denslow JS, Platt WJ, Runkle JR, Spies TA, et al (1990) Light regimes beneath closed canopies and tree-fall gaps in temperate and tropical forests. Can J For Res 20: 620–631 10.1139/x90-084

[7] Hanley TA (2005) Potential management of young-growth stands for understory vegetation and wildlife habitat in southeastern Alaska. Landsc Urban Plan 72: 95–112 10.1016/j.landurbplan.2004.09.015

[8] Frazer GW, Trofymow JA, Lertzman KP (2000) Canopy openness and leaf area in chronosequences of coastal temperate rainforests. Can J For Res 30: 239–256 10.1139/x99-201

[9] Parker G (1995) Structure and microclimate of forest canopies. In: Lowman MD, Nadkarni NM, editors. Forest Canopies. Orlando: Academic Press. 73–98.

[10] Hennon PE, D’Amore DV, Witter DT, Lamb MB (2010) Influence of forest canopy and snow on microclimate in a declining yellow-cedar forest of Southeast Alaska. Northwest Sci 84: 73–87.

[11] Gower ST, Kucharik CJ, Norman JM (1999) Direct and Indirect Estimation of Leaf Area Index, fAPAR, and Net Primary Production of Terrestrial Ecosystems. Remote Sens Environ 70: 29–51.

[12] Jonckheere I, Fleck S, Nackaerts K, Muys B, Coppin P, et al (2004) Review of methods for in situ leaf area index determination: Part I. Theories, sensors and hemispherical photography. Agric For Meteorol 121: 19–35 10.1016/j.agrformet.2003.08.027

[13] Roe AM, Meyer CB, Nibbelink NP, Ben-David M (2010) Differential tree and shrub production in response to fertilization and disturbance by coastal river otters in Alaska. Ecology 91: 3177–3188. 21141179 10.1890/09-1216.1

[14] Welles JM, Cohen S (1996) Canopy structure measurement by gap fraction analysis using commercial instrumentation. J Exp Bot 47: 1335–1342 10.1093/jxb/47.9.1335

[15] Chen JM, Rich PM, Gower ST, Norman JM, Plummer S (2012) Leaf area index of boreal forests: Theory, techniques, and measurements. J Geophys Res 102: 29429–29443.

[16] Decagon Devices (2004) AccuPAR model LP-80. Operator’s manual. Pullman, Washington, USA.

[17] Fassnacht KS, Gower ST, Norman JM, McMurtric RE (1994) A comparison of optical and direct methods for estimating foliage surface area index in forests. Agric For Meteorol 71: 183–207 10.1016/0168-1923(94)90107-4

[18] Hyer EJ, Goetz SJ (2004) Comparison and sensitivity analysis of instruments and radiometric methods for LAI estimation: assessments from a boreal forest site. Agric For Meteorol 122: 157–174 10.1016/j.agrformet.2003.09.013

[19] Leblanc SG, Chen JM (2001) A practical scheme for correcting multiple scattering effects on optical LAI measurements. Agric For Meteorol 110: 125–139 10.1016/S0168-1923(01)00284-2

[20] Stenberg P, Linder S, Smolander H, Flower-Ellis J (1994) Performance of the LAI-2000 plant canopy analyzer in estimating leaf area index of some Scots pine stands. Tree Physiol 14: 981–995. 14967664 10.1093/treephys/14.7-8-9.981

[21] Pierce LL, Running SW (1988) Rapid estimation of coniferous forest leaf area index using a portable integrating radiometer. Ecology 69: 1762–1767 10.2307/1941154

[22] Martens SN, Ustin SL, Rousseau RA (1993) Estimation of tree canopy leaf area index by gap fraction analysis. For Ecol Manag 61: 91–108 10.1016/0378-1127(93)90192-P

[23] McClellan MH (2008) Adaptive management of young stands on the Tongass National Forest. United States Dep Agric For Serv Gen Tech Rep Pnw 733: 225.

[24] Cole EC, Hanley TA, Newton M (2010) Influence of precommercial thinning on understory vegetation of young-growth Sitka spruce forests in southeastern Alaska. Can J For Res 40: 619–628 10.1139/X10-009

[25] Deal RL (2007) Management strategies to increase stand structural diversity and enhance biodiversity in coastal rainforests of Alaska. Biol Conserv 137: 520–532 10.1016/j.biocon.2007.03.014

[26] Hayes JP, Chan SS, Emmingham WH, Tappeiner JC, Kellogg LD, et al (1997) Wildlife response to thinning young forests in the pacific northwest. J For 95: 28–33.

[27] Lindgren PM, Ransome DB, Sullivan DS, Sullivan TP (2006) Plant community attributes 12 to 14 years following precommercial thinning in a young lodgepole pine forest. Can J For Res 36: 48–61 10.1139/x05-228

[28] Carey AB (2000) Effects of new forest management strategieson squirrel populations. Ecol Appl 10: 248–257.

[29] Chan SS, Larson DJ, Maas-Hebner KG, Emmingham WH, Johnston SR, et al (2006) Overstory and understory development in thinned and underplanted Oregon Coast Range Douglas-fir stands. Can J For Res 36: 2696–2711 10.1139/x06-151

[30] Davis LR, Puettmann KJ, Tucker GF (2007) Overstory response to alternative thinning treatments in young Douglas-fir forests of western Oregon. Northwest Sci 81: 1–14 10.3955/0029-344X-81.1.1

[31] Chen JM, Black TA (1992) Defining leaf area index for non-flat leaves. Plant Cell Environ 15: 421–429 10.1111/j.1365-3040.1992.tb00992.x

[32] Frazer GW, Canham CD, Lertzman KP (1999) Gap Light Analyzer (GLA), Version 2.0: Imaging software to extract canopy structure and gap light transmission indices from true-colour fisheye photographs, users manual and program documentation. Simon Fraser Univ Burn Br Columbia Inst Ecosyst Stud Millbrook New York 36. Available: http://rem.sfu.ca/forestry/downloads/Files/GLAV2UsersManual.pdf. Accessed 31 July 2013.

[33] Clark J, Murphy G (2011) Estimating forest biomass components with hemispherical photography for Douglas-fir stands in northwest Oregon. Can J For Res 41: 1060–1074 10.1139/x11-013

[34] Frazer GW, Fournier RA, Trofymow JA, Hall RJ (2001) A comparison of digital and film fisheye photography for analysis of forest canopy structure and gap light transmission. Agric For Meteorol 109: 249–263 10.1016/S0168-1923(01)00274-X

[35] Nobis M, Hunziker U (2005) Automatic thresholding for hemispherical canopy-photographs based on edge detection. Agric For Meteorol 128: 243–250 10.1016/j.agrformet.2004.10.002

[36] Nobis M (2005) SideLook 1.1 - Imaging software for the analysis of vegetation structure with true-colour photographs. http://www.appleco.ch. (Dec 15, 2011).

[37] Gower ST, Grier CC, Vogt DJ, Vogt KA (1987) Allometric relations of deciduous (Larixoccidentalis) and evergreen conifers (Pinuscontorta and Pseudotsugamenziesii) of the Cascade Mountains in central Washington. Can J For Res 17: 630–634 10.1139/x87-103

[38] Helgerson OT, Cromack K, Stafford S, Miller RE, Slagle R (1988) Equations for estimating aboveground components of young Douglas-fir and red alder in a coastal Oregon plantation. Can J For Res 18: 1082–1085 10.1139/x88-164

[39] Tobin B, Black K, Osborne B, Reidy B, Bolger T, et al (2006) Assessment of allometric algorithms for estimating leaf biomass, leaf area index and litter fall in different-aged Sitka spruce forests. Forestry 79: 453–465 10.1093/forestry/cpl030

[40] Turner DP, Acker SA, Means JE, Garman SL (2000) Assessing alternative allometric algorithms for estimating leaf area of Douglas-fir trees and stands. For Ecol Manag 126: 61–76 10.1016/S0378-1127(99)00083-3

[41] Fox J, Weisberg S (2011) An {R} Companion to Applied Regression, Second Edition. Thousand Oaks CA: Sage. Available: http://socserv.socsci.mcmaster.ca/jfox/Books/Companion. Accessed 19 April, 2012.

[42] Zar J (1999) Biostatistical analysis. 4th ed. Upper Saddle River, NJ: Prentice Hall.

[43] R Development Core Team (2011) R: A language and environment for statistical computing. R Foundation for Statistical Computing, Vienna, Austria., http://www.R-project.org/(April 19, 2012).

[44] Schleppi P, Thimonier A, Walthert L (2011) Estimating leaf area index of mature temperate forests using regressions on site and vegetation data. For Ecol Manag 261: 601–610 10.1016/j.foreco.2010.11.013

[45] Planchais I, Pontailler J-Y (1999) Validity of leaf areas and angles estimated in a beech forest from analysis of gap frequencies, using hemispherical photographs and a plant canopy analyzer. Ann For Sci 56: 1–10.

[46] Bréda NJJ (2003) Ground-based measurements of leaf area index: a review of methods, instruments and current controversies. J Exp Bot 54: 2403–2417 10.1093/jxb/erg263 14565947

[47] Inoue A, Yamamoto K, Mizoue N, Kawahara Y (2004) Effects of image quality, size and camera type on forest light environment estimates using digital hemispherical photography. Agric For Meteorol 126: 89–97 10.1016/j.agrformet.2004.06.002

[48] Lang ARG, Yueqin X (1986) Estimation of leaf area index from transmission of direct sunlight in discontinuous canopies. Agric For Meteorol 37: 229–243 10.1016/0168-1923(86)90033-X

[49] Hale SE (2003) The effect of thinning intensity on the below-canopy light environment in a Sitka spruce plantation. For Ecol Manag 179: 341–349 10.1016/S0378-1127(02)00540-6

[50] Grier CG, Running SW (1977) Leaf area of mature northwestern coniferous forests: relation to site water balance. Ecology 58: 893–899.

